# Estimated burden of group a streptococcal pharyngitis among children in Beijing, China

**DOI:** 10.1186/s12879-016-1775-9

**Published:** 2016-08-26

**Authors:** Shuangsheng Wu, Xiaomin Peng, Zuyao Yang, Chunna Ma, Daitao Zhang, Quanyi Wang, Peng Yang

**Affiliations:** 1 Institute for Infectious Disease and Endemic Disease Control, Beijing Center for Disease Prevention and Control, No. 16 Hepingli Middle Street, Dongcheng District Beijing, 100013 China; 2 School of Public Health, Captial Medical University, Beijing, China; 3 Division of Epidemiology, The Jockey Club School of Public Health and Primary Care, The Chinese University of Hong Kong, Hong Kong, SAR China

**Keywords:** Burden, Group A streptococcus, Pharyngitis, China

## Abstract

**Background:**

Burden of Group A streptococcus (GAS) pharyngitis is scarce in developing countries, still unknown in China. The objective of this study was to determine the incidence of clinical cases of pharyngitis and GAS culture-positive pharyngitis, and their outpatient visits among children aged 0–14 years in Beijing, the capital of China.

**Methods:**

Multiplier model was used to estimate the numbers of pharyngitis cases, based on reported numbers of clinical cases and GAS culture-positive rates from GAS surveillances in Beijing, consultation rate, population coverage of GAS surveillances, sampling success rate, and test sensitivity of GAS culture from previous studies, surveys and surveillances.

**Results:**

An average of 29804.6 (95 % CI: 28333.2–31276.0) clinical cases of pharyngitis per 100,000 person-years occurred among children aged 0–14 years, resulting in correspondingly 19519.0 (95 % CI: 18516.7–20521.2) outpatient visits per 100,000 person-years from 2012 to 2014 in Beijing. On average, there were 2685.1 (95 % CI: 2039.6–3330.6) GAS culture-positive cases of pharyngitis and 1652.7 (95 % CI: 1256.5–2049.0) outpatient visits per 100,000 person-years during the same period. The estimated burden of GAS pharyngitis was significantly higher than that of scarlet fever. Children aged 5–14 years had a higher burden of GAS pharyngitis than those aged 0–4 years.

**Conclusions:**

The present data suggests that GAS pharyngitis is very common in children in China. Further studies and surveillances are needed to monitor trends and the effectiveness of control measures.

## Background

Pharyngitis is one of the most common presenting symptom for seeking medications, accounting for an estimated 15 million outpatient visits in 2006 in United States [1]. Group A streptococcus (GAS) is the main bacterial cause of pharyngitis, and responsible for a large number of pharyngitis cases in children [2]. Although GAS pharyngitis may seem relatively benign and unimportant, it causes enormous use of health resources and economic costs [3]. Furthermore, the primary infection can lead to severe GAS diseases (eg, acute rheumatic fever, rheumatic heart disease, post-streptococcal glomerulonephritis, and invasive infection) [4, 5]. A study reviewed recent population-based data and estimated that there were 616 million new GAS pharyngitis cases per year in the world, with an estimated number of more than 1.78 million new cases and 517,000 deaths of severe GAS disease each year [5]. It occurs most commonly among children aged 5–15 years, and its incidence in children varies from region to region [6]. However, epidemiological data on GAS pharyngitis from developing countries is scarce [4–6]. To our knowledge, the disease burden is still unknown in China. In this study, we aimed to determine the incidence of clinical cases of pharyngitis and GAS culture-positive pharyngitis, and their outpatient visits among children aged 0–14 years in Beijing, the capital of China.

## Methods

### GAS surveillances in Beijing

GAS surveillances were conducted in the pediatric clinics of 36 hospitals within Beijing’s 18 districts since May 2011. Since November 2014, the number of sentinel hospitals has been cut down from 36 to 17. The surveillance system was designed and managed by Beijing Center for Disease Prevention and Control. Under the system, clinicians were required to diagnose scarlet fever or pharyngitis and to record the weekly numbers of outpatient visits by age groups (0–4 years, and 5–14 years) on a fixed form. All children with scarlet fever diagnosed by clinicians were invited to participate in the study. Meantime, each week, 10 children with pharyngitis diagnosed by clinicians were randomly selected from each hospital. If there are less than 10 patients in a hospital in a week, all of them were invited to participate in the study. The pharyngeal swab samples were collected by trained clinicians from study participants after their guardians gave informed consent, and tested by the collaborating laboratories. A detailed description of the surveillance was published in a previous study [7].

### National notifiable infectious disease surveillance system in China

Scarlet fever is a notifiable disease according to Law of the People Republic of China on the Prevention and Treatment of Infectious Diseases. All clinical cases of scarlet fever should be reported to the National Notifiable Infectious Disease Surveillance System (NNIDSS) by clinicians and hospitals when they sought medical services in China.

### Case definition

In this study, clinical cases of pharyngitis was defined as follows: pharyngitis is the inflammation of the back of the throat including the tonsils, and its common symptoms include fever, sore throat, red tonsils, and enlarged lymph nodes in the neck and so on. Case definition of clinical cases of scarlet fever conformed to the Diagnostic Criteria for Scarlet Fever (WS282–2008) enacted by the Chinese Ministry of Health [8]. Patients with scarlet fever or pharyngitis from whom GAS was isolated were identified as GAS culture-positive patients.

### Model for cases numbers estimation

**Fig. 1:**
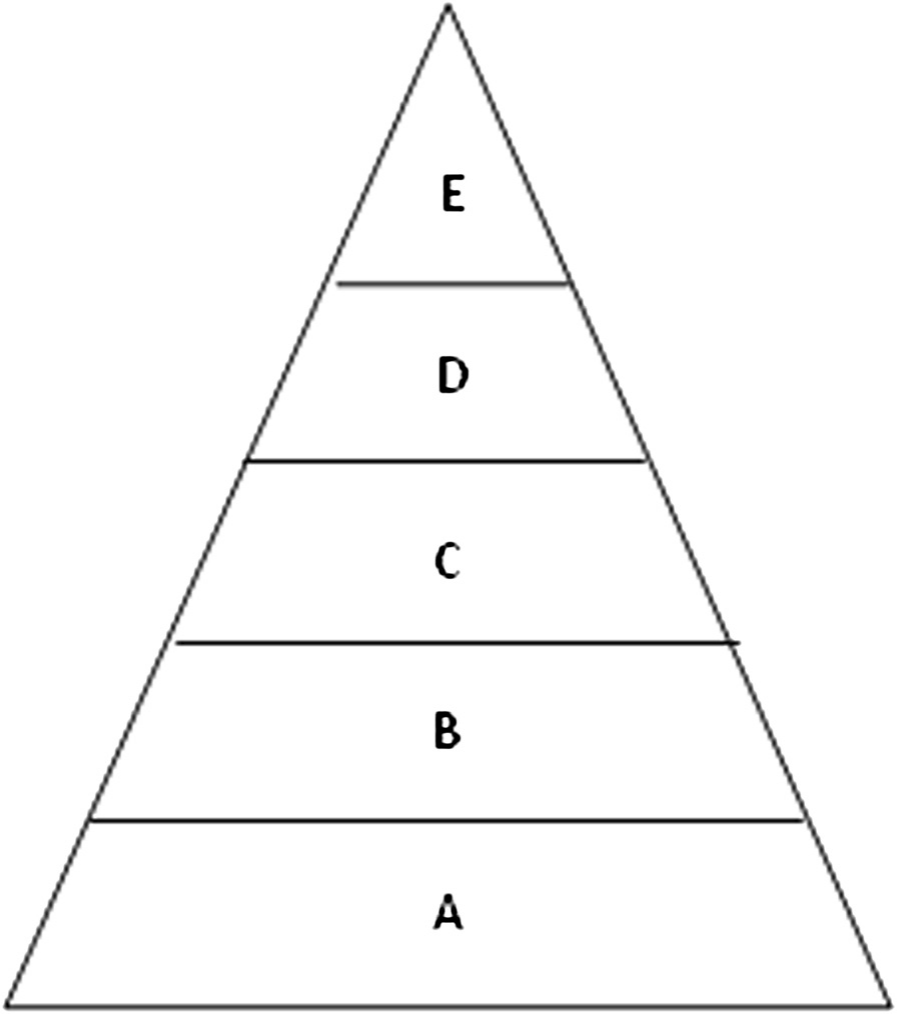
Model parameters for estimating the burden of GAS pharyngitis in Beijing, 2012–2014. Note: A, Total no. cases of GAS culture-positive pharyngitis in Beijing; B, Cases refer to hospitals in Beijing = A* Consultation rate; C, Cases refer to sentinel hospitals of GAS surveillances in Beijing = B*Population coverage of GAS surveillances in Beijing; D, Cases for which specimens were successfully collected = C*Sampling success rate; E, Predicted positive specimens that were correctly identified by laboratory tests = D*Test sensitivity

Previous studies had used the multiplier model to estimate the burden of pandemic (H1N1) 2009 in China and USA [9, 10], and hand, foot and mouth disease in China [11]. In this study, the multiplier model was used to estimate the burden of GAS pharyngitis among children aged 0–14 years in Beijing. To estimate the total number of cases of GAS culture-positive pharyngitis, we built the multiplier model to adjust the count of laboratory-confirmed cases from GAS surveillances in Beijing for the following steps: medical care seeking (B to A), population coverage of GAS surveillances in Beijing (C to B), collection of samples (D to C), and laboratory detection of GAS (E to D) (see Fig. 1). At each step, data for parameter estimations was identified as a range of proportions observed in previous studies, surveys and surveillances.

Because NNIDSS covered all hospitals and all population in Beijing, population coverage of GAS surveillances among scarlet fever cases was calculated by dividing reported number of clinical cases of scarlet fever from GAS surveillances by reported number from NNIDSS in Beijing. In this study, we assumed that population coverage among cases of scarlet fever was equal to that among cases of pharyngitis.

The number of cases of GAS culture-positive pharyngitis was calculated using: (1) Na was age-specific reported number of clinical cases from GAS surveillances. (2) Ra was age-specific GAS culture-positive rate by GAS surveillance in Beijing. (3) Ca was age-specific consultation rate. Consultation rate was calculated by the function (1-proportion of patient who do not visit any health institutions), based on literature data from the Fourth National Health Services Survey of China in 2008, which showed that among children aged 0–4 and 5–14 years, 27.3 and 40.3 % of patients do not visit any health institutions when they were ill [12]. Accordingly, consultation rate among children aged 0–4 and 5–14 years was 72.7 and 59.7 % respectively. (4) Pa, age-specific population coverage of GAS surveillances. (5) s was the success rate for sampling swab samples, ranged from 80 to 90 %, and it was obtained from a study from Beijing [9]. (6) t was the test sensitivity of throat culture, ranged from 75.2 to 97.1 %, and it was obtained from a review study of 13 published literatures [13] (see Eq. 1).

1 $$ \mathrm{Total}\ \mathrm{number}\ \mathrm{of}\ \mathrm{cases}\ \mathrm{of}\ \mathrm{GAS}\ \mathrm{culture}\hbox{-} \mathrm{positive}\ \mathrm{pharyngitis}={\displaystyle \sum \frac{\mathrm{Na}*\mathrm{R}\mathrm{a}}{\mathrm{Ca}*\mathrm{Pa}*\mathrm{s}*\mathrm{t}}} $$

The number of clinical cases of pharyngitis was calculated using: (1) Na, (2) Ca, and (3) Pa (see Eq. 2).

2 $$ \mathrm{Total}\ \mathrm{number}\ \mathrm{of}\ \mathrm{clinical}\ \mathrm{cases}\ \mathrm{of}\ \mathrm{pharyngitis}={\displaystyle \sum \frac{\mathrm{Na}}{\mathrm{Ca}*\mathrm{Pa}}} $$

The number of outpatient visits for GAS culture-positive pharyngitis was calculated using: (1) Na, (2) Ra, (3) Pa, (4) s, and (5) t (see Eq. 3).

3 $$ \mathrm{Total}\ \mathrm{number}\ \mathrm{o}\mathrm{f}\ \mathrm{o}\mathrm{utpatient}\ \mathrm{visits}\ \mathrm{f}\mathrm{o}\mathrm{r}\ \mathrm{GAS}\ \mathrm{culture}\hbox{-} \mathrm{positive}\ \mathrm{pharyngitis}={\displaystyle \sum \frac{\mathrm{Na}*\mathrm{R}\mathrm{a}}{\mathrm{Pa}*\mathrm{s}*\mathrm{t}}} $$

The number of outpatient visits for clinical cases of pharyngitis was a calculation of reported number of clinical cases from GAS surveillances divided by population coverage of GAS surveillances (see Eq. 4).

4 $$ \mathrm{Total}\ \mathrm{number}\ \mathrm{o}\mathrm{f}\ \mathrm{o}\mathrm{utpatient}\ \mathrm{visits}\ \mathrm{f}\mathrm{o}\mathrm{r}\ \mathrm{clinical}\ \mathrm{cases}\ \mathrm{o}\mathrm{f}\ \mathrm{pharyngitis}={\displaystyle \sum \frac{\mathrm{Na}}{\mathrm{Pa}}} $$

The same approach was used to estimate the total number of cases of GAS culture-positive scarlet fever, the total number of clinical cases of scarlet fever, the total number of outpatient visits for GAS culture-positive scarlet fever, and the total number of outpatient visits for clinical cases of scarlet fever.

Annual incidence rate was equal to the estimated number of cases divided by population number from the 2010 National Population Census in Beijing, China [14]. Data from the census showed that there were 1,687,437 persons in the age group of 0–14 in Beijing, of which those aged 0–4 years accounted for 40.65 %.

### Data analysis

Ninety-five percent confidence intervals (CIs) for population coverage and GAS culture-positive rate of GAS surveillances by age groups were calculated using the normal approximation. Difference among the subgroups were tested by Pearson’s Chi-square test with a two-sided *p* value <0.05 as the statistical significance level. Data analyses were carried out using SPSS Version 13.0 (SPSS Inc, Chicago, IL). Ninety-five percent CIs for estimated numbers of cases and annual incidence rate were determined by Monte Carlo simulation, using a multiplier model (Impact 2009, version 1.0) by United States Centers for Disease Control and Prevention.

## Results

### Data from NNIDSS and GAS surveillances in Beijing

The reported numbers of clinical cases of scarlet fever and pharyngitis from GAS surveillances and NNIDSS by years and age groups are shown in Table 1. From 2012 to 2014, a total of 9078 clinical cases of scarlet fever aged 0–14 years were reported from NNIDSS in Beijing, 26.1 % of whom were children aged 0–4 years, and 73.9 % were between the age of 5 and 14 years.

**Table 1:** Reported numbers of clinical cases of scarlet fever and pharyngitis from GAS surveillances and NNIDSS by age groups, Beijing, 2012–2014

Year	Age group	Reported number of clinical cases of scarlet fever from GAS surveillances	Reported number of clinical cases of pharyngitis from GAS surveillances	Reported number of clinical cases of scarlet fever from NNIDSS
2012	0–4	573	79280	998
	5–14	1226	70872	2154
	Overall(0–14)	1799	150152	3152
2013	0–4	286	80595	526
	5–14	712	74356	1503
	Overall(0–14)	998	154951	2029
2014	0–4	299	71132	842
	5–14	997	70997	3055
	Overall(0–14)	1296	142129	3897
2012–2014	0–4	1158	231007	2366
	5–14	2935	216225	6712
	Overall(0–14)	4093	447232	9078

During the same period, a total of 4093 clinical cases of scarlet fever and 447,232 ones of pharyngitis were reported from GAS surveillances in Beijing. Of the 4093 clinical cases of scarlet fever, 28.3 % were children aged 0–4 years and 71.7 % were between the age of 5 and 14 years. Of the 447,232 clinical cases of pharyngitis, 51.7 % were children aged 0–4 years and 48.3 % were between the age of five and fourteen years.

### Population coverage of GAS surveillances in Beijing

Using the surveillance data from NNIDSS and GAS surveillances, we estimated the population coverage of GAS surveillances among children aged 0–4 years and 5–14 years in Beijing, respectively. Because the number of sentinel hospitals was cut down from 36 to 17, the population coverage of GAS surveillances in 2014 was significantly lower than that in 2012 and 2013 (see Table 2).

**Table 2:** Estimates of population coverage of GAS surveillances by age groups, Beijing, 2012–2014

Year	Age group	Reported number of clinical cases of scarlet fever from GAS surveillances	Reported number of clinical cases of scarlet fever from NNIDSS	Population coverage of GAS surveillances		
				Proportion (%)	95 % CI	
2012	0–4	573	998	57.4	54.3	60.5
	5–14	1226	2154	56.9	54.8	59.0
	Overall(0–14)	1799	3152	57.1	55.3	58.8
2013	0–4	286	526	54.4	50.1	58.6
	5–14	712	1503	47.4	44.8	49.9
	Overall(0–14)	998	2029	49.2	47.0	51.4
2014	0–4	299	842	35.5	32.3	38.7
	5–14	997	3055	32.6	31.0	34.3
	Overall(0–14)	1296	3897	33.3	31.8	34.7

### GAS culture-positive rate of GAS surveillances in Beijing

Bacterial surveillance data for GAS showed that the overall positive rate of GAS causing scarlet fever was significantly higher than pharyngits during the 3 years (*p* < 0.05). The lower rate of GAS causing pharyngits in 2013 compared to 2012 and 2014 was statistically significant (*p* < 0.05), but no significant difference of positive rate of GAS causing scarlet fever was found between the 3 years (*p* > 0.05). With respect to the difference between the two age groups, we observed higher positive rate of GAS causing pharyngits among children aged 5–14 years in all the 3 years (*p* < 0.05). Nonetheless, no significant association between age and positive rate of GAS causing scarlet fever existed in any of 3 years (*p* > 0.05) (see Table 3).

**Table 3:** GAS culture-positive rate of GAS surveillances in Beijing by age groups, Beijing, 2012–2014

Year	Clinical manifestations	Age group	Samples no.	GAS culture-positive rate		
				Proportion (%)	95 % CI	
2012	Scarlet fever	0–4	71	45.1	33.5	56.6
		5–14	154	53.2	45.4	61.1
		Overall(0–14)	225	50.7	44.1	57.2
	Pharyngitis	0–4	1012	3.0	1.9	4.0
		5–14	906	14.9	12.6	17.2
		Overall(0–14)	1918	8.6	7.3	9.9
2013	Scarlet fever	0–4	50	36.0	22.7	49.3
		5–14	110	39.1	30.0	48.2
		Overall(0–14)	160	38.1	30.6	45.7
	Pharyngitis	0–4	1382	1.0	0.5	1.5
		5–14	1295	4.6	3.4	5.7
		Overall(0–14)	2677	2.7	2.1	3.3
2014	Scarlet fever	0–4	50	42.0	28.3	55.7
		5–14	216	43.5	36.9	50.1
		Overall(0–14)	266	43.2	37.3	49.2
	Pharyngitis	0–4	1479	2.3	1.5	3.1
		5–14	1472	11.4	9.8	13.0
		Overall(0–14)	2951	6.8	5.9	7.8

### Numbers of clinical cases and GAS culture-positive cases

Using the multiplier model, we estimated that there were 398,720 clinical cases of pharyngitis in 2012, 467,670 cases in 2013 and 642,410 cases in 2014, with an annual incidence rate of 23628.7, 27714.8 and 38070.2 cases per 100,000 children, respectively. Of these clinical cases, a total of 50,499, 19,258, and 66,172 pharyngitis cases were GAS culture-positive during 2012 to 2014, with an annual incidence rate of 2992.6, 1141.3 and 3921.5 cases per 100,000 children, respectively. The incidence of GAS culture-positive pharyngitis was statistically significantly lower in 2013 than in 2012 and 2014 (*p* < 0.05), but no significant difference was found between 2012 and 2014 (*p* > 0.05). With respect to the difference between the two age groups, we observed a higher risk for incidence of GAS culture-positive pharyngitis among children aged 5–14 years in all the 3 years (4273.5 vs. 1122.2 cases per 100,000 children in 2012, 1636.4 vs. 418.2 cases per 100,000 children in 2013, and 5728.5 vs. 1282.8 cases per 100,000 children in 2014, *p* < 0.05). The annual incidence rate of GAS culture-positive pharyngitis was more than 10 times higher than that of scarlet fever during the 3 years (2992.6 vs. 206.9 cases per 100,000 children in 2012, 1141.3 vs. 101.7 cases per 100,000 children in 2013, and 3921.5 vs. 220.6 cases per 100,000 children in 2014, *p* < 0.05) (see Table 4).

**Table 4:** Estimated numbers of clinical cases and GAS culture-positive cases, Beijing, 2012–2014

Year	Clinical manifestations	Age groups	Clinical cases						GAS culture-positive cases					
			*n*	95 % CI		Annual incidence rate	95 % CI		*n*	95 % CI		Annual incidence rate	95 % CI	
2012	Scarlet fever	0–4	1374.5	1289.8	1459.2	200.4	188.0	212.8	848.8	558.4	1139.3	123.8	81.4	166.1
		5–14	3607.6	3452.8	3762.4	360.2	344.7	375.7	2642.1	2003.3	3280.9	263.8	200.0	327.6
		Overall(0–14)	4982.1	4806.0	5158.2	295.2	284.8	305.7	3490.9	2712.2	4269.6	206.9	160.7	253.0
	Pharyngitis	0–4	190170.0	178448.6	201891.4	27726.4	26017.5	29435.4	7696.7	4258.0	11135.4	1122.2	620.8	1623.5
		5–14	208550.0	199602.5	217497.5	20822.6	19929.2	21715.9	42802.0	32187.9	53416.1	4273.5	3213.8	5333.3
		Overall(0–14)	398720.0	384004.8	413435.2	23628.7	22756.7	24500.8	50499.0	38576.9	62421.1	2992.6	2286.1	3699.2
2013	Scarlet fever	0–4	725.1	660.7	789.5	105.7	96.3	115.1	360.6	199.1	522.1	52.6	29.0	76.1
		5–14	2521.7	2368.5	2674.9	251.8	236.5	267.1	1355.8	920.0	1791.6	135.4	91.9	178.9
		Overall(0–14)	3246.7	3080.6	3412.8	192.4	182.6	202.2	1716.4	1213.6	2219.2	101.7	71.9	131.5
	Pharyngitis	0–4	204320.0	186174.5	222465.5	29789.5	27143.9	32435.0	2868.5	1098.3	4638.7	418.2	160.1	676.3
		5–14	263340.0	247341.8	279338.2	26293.1	24695.7	27890.4	16389.0	10957.0	21821.0	1636.4	1094.0	2178.7
		Overall(0–14)	467670.0	443493.5	491846.5	27714.8	26282.1	29147.6	19258.0	13397.0	25119.0	1141.3	793.9	1488.6
2014	Scarlet fever	0–4	1166.2	1043.0	1289.4	170.0	152.1	188.0	678.0	396.4	959.7	98.9	57.8	139.9
		5–14	5125.4	4829.3	5421.5	511.7	482.2	541.3	3044.8	2316.5	3773.1	304.0	231.3	376.7
		Overall(0–14)	6291.5	5974.1	6608.9	372.8	354.0	391.7	3722.8	2881.0	4564.6	220.6	170.7	270.5
	Pharyngitis	0–4	277430.0	248129.5	306730.5	40448.8	36176.8	44720.7	8798.4	5111.4	12485.4	1282.8	745.2	1820.4
		5–14	364980.0	343892.7	386067.3	36441.3	34335.8	38546.7	57374.0	43707.7	71040.3	5728.5	4364.0	7093.0
		Overall(0–14)	642410.0	606815.7	678004.3	38070.2	35960.8	40179.5	66172.0	51279.1	81064.9	3921.5	3038.9	4804.0
Average: 2012−2014	Scarlet fever	0−4	1088.6	997.8	1179.3	158.7	145.5	171.9	629.2	384.6	873.7	91.7	56.1	127.4
		5−14	3751.6	3550.2	3952.9	374.6	354.5	394.7	2347.6	1746.6	2948.5	234.4	174.4	294.4
		Overall(0–14)	4840.1	4620.2	5060.0	286.8	273.8	299.9	2976.7	2268.9	3684.5	176.4	134.5	218.3
	Pharyngitis	0−4	223973.3	204250.9	243695.8	32654.9	29779.4	35530.4	6454.5	3489.2	9419.8	941.1	508.7	1373.4
		5−14	278956.7	263612.3	294301.0	27852.3	26320.2	29384.4	38855.0	28950.9	48759.1	3879.5	2890.6	4868.3
		Overall(0–14)	502933.3	478104.7	527762.0	29804.6	28333.2	31276.0	45309.7	34417.7	56201.7	2685.1	2039.6	3330.6

### Outpatient visits for clinical cases and GAS culture-positive cases

As shown in Table 5, we estimated that there were 262,760, 305,760, and 419,590 outpatient visits for clinical cases of pharyngitis during 2012 to 2014, with an annual incidence rate of 15571.5, 18119.8 and 24865.5 cases per 100,000 children, respectively. Of these outpatient visits for clinical cases, a total of 31,148, 11,870 and 40,648 cases of pharyngitis were GAS culture-positive during the same period, with an annual incidence rate of 1845.9, 703.4, and 2408.9 cases per 100,000 children, respectively. The incidence of outpatient visits for GAS culture-positive pharyngitis was statistically significantly lower in 2013 than in 2012 and 2014 (*p* < 0.05), but no significant difference was found between 2012 and 2014 (*p* > 0.05). Compared to children aged 0–4 years, those aged 5–14 years had a higher risk of outpatient visits for GAS culture-positive pharyngitis in all the 3 years (2551.3 vs. 815.8 cases per 100,000 children in 2012, 976.9 vs. 304 cases per 100,000 children in 2013, and 3419.9 vs. 932.6 cases per 100,000 children in 2014, *p* < 0.05). The incidence of outpatient visits for GAS culture-positive pharyngitis was significantly higher than that of scarlet fever during the 3 years (1845.9 vs. 130 cases per 100,000 children in 2012, 703.4 vs. 231.6 cases per 100,000 children in 2013, and 2408.9 vs. 136.9 cases per 100,000 children in 2014, *p* < 0.05) (see Table 5).

**Table 5:** Estimates of outpatient visits for clinical cases and GAS culture-positive cases, Beijing, 2012−2014

Year	Clinical manifestations	Age groups	Clinical cases						GAS culture−positive cases					
			*n*	95 % CI		Annual incidence rate	95 % CI		*n*	95 % CI		Annual incidence rate	95 % CI	
2012	Scarlet fever	0−4	999.3	937.7	1060.8	145.7	136.7	154.7	617.1	405.9	828.3	90.0	59.2	120.8
		5−14	2153.7	2061.3	2246.1	215.0	205.8	224.3	1577.3	1195.9	1958.7	157.5	119.4	195.6
		Overall(0–14)	3153.0	3042.2	3263.8	186.9	180.3	193.4	2194.4	1705.0	2683.8	130.0	101.0	159.0
	Pharyngitis	0−4	138260.0	129738.6	146781.4	20158.0	18915.6	21400.5	5595.5	3095.6	8095.4	815.8	451.3	1180.3
		5−14	124500.0	119158.3	129841.7	12430.6	11897.3	12964.0	25553.0	19216.4	31889.6	2551.3	1918.7	3184.0
		Overall(0–14)	262760.0	252722.4	272797.6	15571.5	14976.7	16166.4	31148.0	23795.2	38500.8	1845.9	1410.1	2281.6
2013	Scarlet fever	0−4	527.1	480.3	573.9	76.9	70.0	83.7	262.2	144.8	379.5	38.2	21.1	55.3
		5−14	1505.4	1413.9	1596.9	150.3	141.2	159.4	809.4	549.2	1069.6	80.8	54.8	106.8
		Overall(0–14)	2032.6	1929.9	2135.3	120.5	114.4	126.5	1071.6	759.5	1383.7	63.5	45.0	82.0
	Pharyngitis	0−4	148540.0	135348.2	161731.8	21656.8	19733.5	23580.2	2085.4	798.4	3372.4	304.0	116.4	491.7
		5−14	157220.0	147669.1	166770.9	15697.6	14744.0	16651.2	9784.3	6541.4	13027.2	976.9	653.1	1300.7
		Overall(0–14)	305760.0	289483.0	322037.0	18119.8	17155.2	19084.4	11870.0	8276.2	15463.8	703.4	490.5	916.4
2014	Scarlet fever	0−4	847.8	758.3	937.3	123.6	110.6	136.7	492.9	288.2	697.7	71.9	42.0	101.7
		5−14	3059.8	2883.0	3236.6	305.5	287.9	323.2	1817.7	1382.9	2252.5	181.5	138.1	224.9
		Overall(0–14)	3907.6	3711.8	4103.4	231.6	220.0	243.2	2310.6	1787.2	2834.0	136.9	105.9	167.9
	Pharyngitis	0−4	201690.0	180388.5	222991.5	29406.0	26300.3	32511.7	6396.4	3715.9	9076.9	932.6	541.8	1323.4
		5−14	217890.0	205300.9	230479.1	21755.1	20498.2	23012.1	34252.0	26093.2	42410.8	3419.9	2605.3	4234.5
		Overall(0–14)	419590.0	395166.5	444013.5	24865.5	23418.1	26312.9	40648.0	31534.6	49761.4	2408.9	1868.8	2948.9
Average: 2012−2014	Scarlet fever	0−4	791.4	725.4	857.4	115.4	105.8	125.0	457.4	279.6	635.2	66.7	40.8	92.6
		5−14	2239.6	2119.4	2359.8	223.6	211.6	235.6	1401.5	1042.7	1760.3	139.9	104.1	175.8
		Overall(0–14)	3031.1	2894.6	3167.5	179.6	171.5	187.7	1858.9	1417.2	2300.5	110.2	84.0	136.3
	Pharyngitis	0−4	162830.0	148491.8	177168.2	23740.3	21649.8	25830.8	4692.4	2536.7	6848.2	684.1	369.8	998.5
		5−14	166536.7	157376.1	175697.3	16627.8	15713.1	17542.4	23196.4	17283.7	29109.2	2316.0	1725.7	2906.4
		Overall(0–14)	329370.0	312457.3	346282.7	19519.0	18516.7	20521.2	27888.7	21202.0	34575.3	1652.7	1256.5	2049.0

## Discussion

This is the first study to estimate the incidence of clinical cases of pharyngitis and GAS culture-positive pharyngitis among children in China. It was estimated that an average of 502933.3 clinical cases of pharyngitis occurred in children aged 0–14 years with about 29.8 (95 % CI: 28.3–31.3) cases per 100 child-years in Beijing, from 2012 to 2014. Although pharyngitis may seem relatively benign and unimportant, our study found that it caused as many as 329,370 outpatient visits each year with about 19.5 (95 % CI: 18.5–20.5) cases per 100 child-years in Beijing during 2012 to 2014, which indicated enormous use of health resources and medical costs. Our results are consistent with data from the National Ambulatory Medical Care Survey in United States, which reported that pharyngitis are responsible for 20 visits to a physician per 100 population annually in the United States [15]. These data highlighted that pharyngitis is a very common presenting symptom for seeking medications among children in Beijing, China.

In our study, we estimated that an average of 45309.7 pharyngitis cases were laboratory confirmed per year from 2012 to 2014 in Beijing, with an annual incidence rate of 2.7 (95 % CI: 2.0–3.3) per 100 person-years for children, respectively, similar to the rate of GAS pharyngitis reported from a study in Europe (3.9 per 100 persons per year) [16], but lower than recently reported rates in Australia (13 per 100 person-years in 2001/2002 [17], and 14 per 100 person-years in 2001 [18]), and Fiji (14.7 per 100 person-years in 2006 [19]). In a peri-urban population of northern India, the incidence of GAS pharyngitis among 5–15-year-old schoolchildren, with an estimation of 95 per 100 person-years in 1995/1996 [20], was significantly higher than our estimation in Beijing. There were two reasons which may partially explain the lower rates of GAS pharyngitis in Beijing. First, GAS-related diseases are highly transmissible within populations characterized by crowding, limited access to hygiene and inadequate medical care [20]. As one of the most developed regions in China, Beijing has undergone epidemiological transition from communicable to non-communicable diseases as the predominant causes of morbidity and mortality. Scarlet fever is usually used as a proxy of GAS-related diseases because of its distinctive clinical features. During the period from 1949 to 2006, the incidence of scarlet fever has remarkably fallen from 488.3 to 1.86 cases per 100000 persons among all age population in Beijing [21]. Second, although immunity to GAS is emm-type specific [22], most of GAS samples from Beijing GAS surveillances were indentified as emm-1 and 12 types, from 2011 to 2014 [23]. Therefore, a large percentage of Beijing population had become immune to GAS infection from 2012 to 2014 because of the 2011 GAS epidemic in Beijing [7].

GAS causes a broad spectrum of diseases, ranging from mild superficial infections of the throat or skin to severe invasive infections and the post-streptococcal complications of acute rheumatic fever and acute post-streptococcal glomerulonephritis. In China, scarlet fever is the only notifiable disease among these GAS diseases according to the Law, but other GAS related infections are not notifiable and there are very few active surveillance systems. In our study, we found that the incidence of GAS pharyngitis was more than 10 times higher than that of scarlet fever. Accordingly, GAS pharyngitis caused more outpatient visits than GAS scarlet fever among children in Beijing. These results indicated that the disease burden of GAS pharyngitis has been significant, and posing great threats to the health of children in Beijing. Therefore, more epidemiological studies and surveillance of other GAS related infections should be developed in Beijing, and other regions in China.

Our result demonstrated that the incidence of GAS pharyngitis among children aged 5–15 years was heavier than in preschool children aged 0–4 years due to the distinct difference of GAS culture-positive rates across the two agegroups. The result confirmed that disease burden of GAS is higher from 5–15 years than with younger children. However, the GAS culture-positive rates of scarlet fever didn’t vary smililarly. Unlike clinical cases of pharyngitis, clinical cases of scarlet fever had more distinctive clinical features and specific case definition in this study. Thus it was easily for clinicians to diagnose these cases without laboratory tests. Accordingly, the GAS culture-positive rate of scarlet fever was much higher than that of pharyngitis in all agegroups, and only a small difference was observed between the two agegroups.

In this study, we observed the significant variation in yearly incidence, which may be explained by epidemic period of GAS. As a proxy of GAS-related diseases, epidemiological data showed that the epidemic period of scarlet fever was about 6–8 years in Beijing [21]. The factors including genetic variation, environmental factors, and host immune status might have contributed to the epidemic period [7]. But further studies should be conducted to analyse these factors.

Our study has several limitations. First, because pharyngitis was not a notifiable disease in Beijing, some records for pharyngitis outpatient visits might be omitted by clinicians when they were busy to diagnose and treat the patients. Therefore, the estimated incidence for GAS pharyngitis in our study might have been underestimated. Second, there was no survey of health-seeking behavior among patients of GAS pharyngitis, so we have to use the age-specific consultation rate of all diseases from the Fourth National Health Services Survey of China (NHSS) in 2008. Nevertheless, the consultation rate of patient-defined Hand, Foot, and Mouse disease was estimated at 75.1 % among children under 5 years of age in Beijing, which is almost equal to the rate of all diseases at 72.7 % from NHSS [11]. The finding may indicate that the consultation rates for parameter estimations were acceptable and reliable. Third, as a model study, additional data from surveys of health-seeking behavior among patients of GAS diseases and reporting quality of physician practice could help refine the parameter estimates [10]. Although the multiplier model provided a quicker and more representative results than population-based surveys, a prospective, cohort study in China is needed to confirm our estimations since the model has not been used to estimate GAS burden.

## Conclusions

In conclusion, we estimated an average of 29.8 clinical cases of pharyngitis and 2.7 cases of GAS culture-positive pharyngitis per 100 person-years among children aged 0–14 years, resulting in a large number of outpatient visits from 2012 to 2014 in Beijing, China. These estimates suggest that the disease burden of GAS pharyngitis has been significant, and posing great threats to the health of children in China. More epidemiological studies and surveillances should be developed to estimate the burden of GAS related diseases in Beijing, and other regions in China.
